# Ethnobotanical study of wild edible plants of Kara and Kwego semi-pastoralist people in Lower Omo River Valley, Debub Omo Zone, SNNPR, Ethiopia

**DOI:** 10.1186/1746-4269-6-23

**Published:** 2010-08-17

**Authors:** Tilahun Teklehaymanot, Mirutse Giday

**Affiliations:** 1 Aklilu Lemma Institute of Pathobiology, Addis Ababa University, P. O. Box 1176, Addis Ababa, Ethiopia

## Abstract

**Background:**

The rural populations in Ethiopia have a rich knowledge of wild edible plants and consumption of wild edible plants is still an integral part of the different cultures in the country. In the southern part of the country, wild edible plants are used as dietary supplements and a means of survival during times of food shortage. Therefore, the aim of this study is to document the wild edible plants gathered and consumed by Kara and Kwego people, and to analyze patterns of use between the two people.

**Methods:**

A cross sectional ethnobotanical study of wild edible plant species was conducted from January 2005 to March 2007. About 10% of each people: 150 Kara and 56 Kwego were randomly selected to serve as informants. Data were collected using semi-structured questionnaire and group discussions. Analysis of variance (α = 0.05) was used to test the similarity of species richness of wild edible plants reported by Kara and Kwego people; Pearson's Chi-square test (α = 0.05) was used to test similarity of growth forms and plant parts of wild edible plants used between the two people.

**Results:**

Thirty-eight wild plant species were reported as food sources that were gathered and consumed both at times of plenty and scarcity; three were unique to Kara, five to Kwego and 14 had similar local names. The plant species were distributed among 23 families and 33 genera. The species richness: families, genera and species (p > 0.05) were not significantly different between Kara and Kwego. Nineteen (50%) of the reported wild edible plants were trees, 11 (29%) were shrubs, six (16%) were herbs and two (5%) were climbers. Forty plant parts were indicated as edible: 23 (58.97%) fruits, 13 (33.33%) leaves, 3 (7.69%) roots and one (2.56%) seed. There was no difference between wild edible plants growth forms reported (Pearson's Chi-square test _(d.f. = 3) _= 0.872) and plant parts used (Pearson's Chi-square test _(d.f. = 3) _= 0.994) by Kara and Kwego people. The majority of wild edible plants were gathered and consumed from *'Duka' *(March) to *'Halet' *(May) and from *'Meko' *(August) to *'Tejo' *(November). Sixteen (41%) of the plant parts were used as a substitute for cultivated vegetables during times of scarcity. The vegetables were chopped and boiled to make *'Belesha' *(sauce) or as a relish to *'Adano' *(porridge). The ripe fruits were gathered and consumed fresh and some were made into juices. The seeds and underground parts were only consumed in times of famine. Thirty-seven percent of the wild edible plants were used as medicine and 23.6% were used for other functions.

**Conclusions:**

The wild edible plants were used as supplements to the cultivated crops and as famine foods between harvesting seasons. But information on the nutritional values and possible toxic effects of most of the wild edible plants reported by Kara and Kwego, and others in different part of Ethiopia is not available. Therefore, the documented information on the wild edible plants may serve as baseline data for future studies on nutritional values and possible side effects, and to identify plants that may improve nutrition and increase dietary diversity. Some of these wild edible plants may have the potential to be valuable food sources (if cultivated) and could be part of a strategy in tackling food insecurity.

## Background

Utilization of wild edible plants as a food source is an integral part of the culture of indigenous people that dwell in the rain forests of Africa and South America [1-4] who gather and consume wild edible plants as snacks and at times of food scarcity [5-10]. Sixteen indigenous people inhabit the south western part of Ethiopia; Kara and Kwego are two of these that live in Lower Omo River Valley. They are knowledgeable of traditional plant uses, one of which is the use of wild edible plants as a food source [10-13].

The Kara people live in the remote section of the Lower Omo Valley on the eastern bank of Omo River. They have a well-kept and distinct cultural identity, and speak the Omotic language. The Kara people use ornate body art, intricate headdresses and body scarification to express beauty and significance in the community. They are known by their painted body and face decorations, which are symbolic and ornamental expression. The body painting is an elaborate process, which ranges from fine and elaborate details to rough, but striking paintings traced with the palms or fingers that combines white (chalk), black (charcoal), yellow, ochre, and red earth. They are semi-pastoralists and fishermen [11,13].

The Kwego people live on the western bank of the Omo River at its junction with the Mago River. They live in a symbiotic relationship with the Kara people and speak Nilotic language, which is threatened by the influence of Bumea people. They are hunter-gatherers and fishermen. They also grow sorghum and maize on the shore of Omo River and collect honey from wild [11,13].

The Ethiopian flora contains approximately 6000 species of higher plants of which about 10% are endemic [14,15]. It is known as a 'biodiversity hot-spot' and a centre of origin and diversification for a significant number of food plants and their wild relatives [16,17]. The rural populations in Ethiopia have a rich knowledge of wild edible plants and consumption of wild edible plants is still an integral part of the different cultures in country. Ethnobotanical studies conducted in Ethiopia have indicated that over 300 species of wild plants are gathered and consumed by the people [8,10,17-22].

In the southern part of Ethiopia, wild edible plants are used as supplements to cultivated crops and as a survival strategy during food shortages that appears to have been intensified due to low development of agriculture and the repeated lack of rain [10,12,19]. Nevertheless, the Kara and Kwego people have subsisted primarily on pastoralism and agriculture and therefore, still preserve traditional knowledge on wild edible plants and depend on these plant species in most of the seasons where food shortages occur: *'Leamura' *(July) to *'Kilikila' *(December). Although the rich indigenous knowledge on the medicinal use of plants has been relatively well documented [8,9], studies on the knowledge of wild edible plants in Ethiopia are limited [10,23]. The aim of this study is to document the use of wild edible plants gathered and consumed by Kara and Kwego people, and analyse patterns of use between the two people. The results may be useful to identify wild edible plants that can improve nutrition and increase dietary diversity in a similar environment. Some of these wild edible plants may also have the potential to be valuable food sources (if cultivated) and so be an important strategy in tackling food insecurity.

## Methods

### Study sites

**Figure 1:**
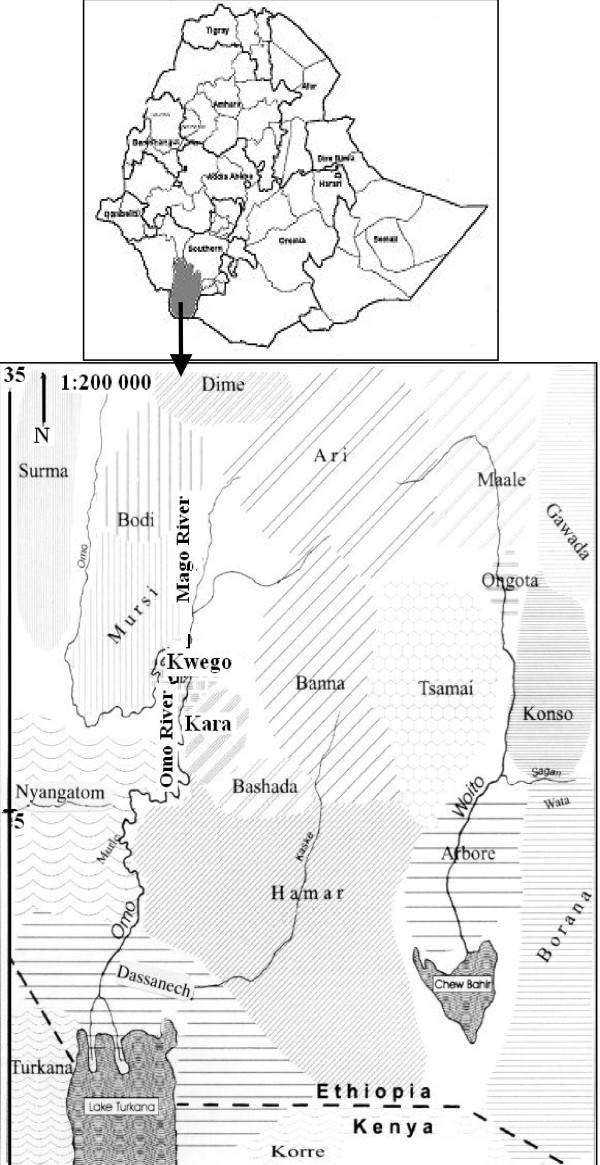
**Map of the study area in Debub Omo Zone, Southern Nations, Nationalities and Peoples Regional State, Ethiopia**.

Kara and Kwego are located in the Lower Omo River Valley, Debub Omo Zone, Southern Nations, Nationalities and Peoples Regional State (SNNPR) at about 880 km south of Addis Ababa. The study site is *'kola' *to *'bereha' *(dry arid lowland), has 400 - 600 mm mean annual rainfall and 20.1 - 27.5°C mean annual temperature. The Kara people live in three villages: Lebok (403.26 m.a.s.l., N 050 22' 306'', E 360 12' 575''), Duss (406.30 m.a.s.l., N 050 16' 471'', E 360 12' 420'') and Korcho (433.13 m.a.s.l., N 050 11' 562', E 360 12' 428'') on the eastern bank of Omo River in Hamar Woreda (District). They are bordering Hamar and Bena on the east, Nyangatom on the west, Mursi on the north, and Dasenech on the south. The total population of the three villages is 1,472 (Fig. 1).

The Kwego people live at six villages on the western bank of Omo River in Kuraz Woreda and the total population is 584. The location with the highest population and the prominent residential area is Kuchuru (406 m.a.s.l., N 050 25' 683'', E 360 12' 717''), which is located at about 42 km from Kangaton. They live together with Bumea/Nyangatom people. They are bordering Nyangatom on the south and west, Mursi on the north and Kara on the east (Fig. 1).

### Ethnobotanical data collection

A cross sectional ethnobotanical study was conducted from January 2005 to March 2007. About 10% of each people: 150 Kara (female = 48, male = 112) and 56 Kwego (female = 20, male = 36) were randomly selected using random numbers, which were generated using Microsoft Excel 2003, from households at each location to serve as informants: The Kara female informants' age range was from 20 years to 70 years (median = 43) and that of the males was from 23 years to 80 years (median = 58.5). The Kwego females' age ranged from 20 years to 80 years (median = 29.5) and that of males' from 20 years to 85 years (median = 45) [24]. The data on wild edible plants were collected using semi-structured questionnaire and group discussions. The group discussions were used to elaborate on time of farming, food scarcity and gathering, and mode of consumptions. Interviews and discussions were conducted with the assistance of native translators. Information on local names of the plants, parts of plants used, mode of consumption, preparation and time of gathering were recorded. Voucher specimens were collected during walks with informants. Plants were identified by experts, Meleaku Wondafrash, at the National Herbarium of Addis Ababa University and Aklilu Lemma Institute of Pathobiology (ALIPB). Voucher specimens were deposited at ALIPB, Addis Ababa University.

### Data analysis

Percentage, and bar and pie charts were used to summarize the data. Analysis of variance (α = 0.05) was used to test the similarity of the wild edible plant species richness reported by Kara and Kwego people; Pearson's Chi-square test (α = 0.05) was used to test similarity of growth forms and plant parts of wild edible plant species used as food source between Kara and Kwego people. The statistical software used was SPSS for Windows, Rel. 11.0.1. 2001. Chicago: SPSS Inc.

### Ethical consideration

The study was ethically approved by the Ethical Committee of Aklilu Lemma Institute of Pathobiology, Addis Ababa University. Prior to the collection of data, permission was secured from the Debub Omo Zone Administration and verbal consent was obtained from the informants, Kara and Kwego people, after elaborating the aim of the study with assistance of native translators.

## Results and discussion

### Taxonomic diversity

Thirty-eight wild plant species were reported as sources of food by Kara and Kwego informants; three were unique to Kara, five to Kwego and fourteen had similar local names (Table 1). The taxonomic richness (families, genera and species, p > 0.05, Table 2) was not significantly different between Kara and Kwego people. The two people live in symbiotic relationship; share a similar type of ecology and knowledge of wild edible plant uses. The Kara and Kwego people live in a semi arid and arid region [13,19,25]. The total number of wild edible plants reported by Kara and Kwego informants was pooled together in the analysis of the distribution of the species.

**Table 1:** List of wild edible plants reported by Kara and Kwego people

Family	Species	Local Name	Habit	Plant part used	Preparation	Voucher Number
Annonaceae	*Uvaria leptocladon *Oliv.	Chochum (KW) Zebko (KA)	Tree	Fruit (whole)	Ripe and row	MUG-25
Apocynaceae	*Saba comorensis *(Boj.) Pichon	Goriza (KA) Geri (KW)	Tree	Fruit (whole)	Row (juice) or boiled	DUS-11
Araceae	*Borassus aethiopum *Mart.	Gullo	Tree	Fruit (exocarp)	Ripe and raw	DUS-4
Asclepiadaceae	*Leptadenia hastala *(Pers.) Decne.	Surmudo	Shrub	Leaves/young shoot	Vegetable, boiled	KOR-40
Balanitaceae	*Balanites rotundifolia *(Van Tiegh.) Blatter	Moroko	Tree	Fruit (whole)	Ripe and raw, famine food	MUG-40
Boraginaceae	*Cordia sinensis *Lam.	Midir/Togoz (KA) Chuwacho (KW)	Tree	Fruit (whole)	Raw, famine food	MUR-35
	*Heliotropium steudneri *Vatke	Gabo	Tree	Fruit (excluding peel)	Raw	DUS-60
Capparidaceae	*Cadaba forinosa *Forssk.	Diri (KA) Meti (KW)	Shrub	Leaf	Vegetable, boiled	MUR-44
	*Cleome gynandra *L.	Iresa	Shrub	Leaf	Vegetable, boiled	LEB-20
	*Maerua oblongifolia *(Forssk.) A. Rich.	Lecho	Shrub	Leaf	Vegetable, boiled	DUS-2
	*Maerua subcordata *(Gilg.) De Wolf	Kulup	Tree	Fruit (excluding peel)	Ripe and raw	DUS-8
Celasteraceae	*Maytenus senegalensis *(Lam.) Exell	Lele (KW)	Tree	Leaf	Vegetable, boiled	DUS-24
Convolvulaceae	*Convolvulus glomeratus *Choisy	Bolok (KW)	Herb	Leaf	Vegetable, boiled	MUG-20
	*Ipomoea plebeia *R. Br.	Boloko (KA)	Shrub	Leaf	Vegetable, boiled	LEB-22
Cucurbitaceae	*Coccinia grandis *(L.) Voigt	Buta (KA)	Climber	Fruit (whole)	Vegetable, boiled	LEB-17
	*Kedrostis foetidissma *(Jacq.) Cogn.	Shunto	Climber	Leaf	Vegetable, boiled	LEB-06
Ebenaceae	*Diospyros mespiliformis *Hochst.ex A. DC.	Kerenso (KW)	Tree	Fruit (whole)	Vegetable, boiled	MUR-36
Euphorbiaceae	*Flueggea virosa *(Willd.) Voigt.	Tanta (KA)	Tree	Fruit (whole)	Vegetable, famine food	LEB-19
Fabaceae	*Fabaceae sp*.	Torowo (KA) Quamu (KW)	Herb	Leaf or young shoot	Vegetable boiled	KOR-4
	*Tamarindus indica *L.	Roka (KA) Raku (KW)	Tree	Fruit (whole)	Raw (juice) or boiled famine food	DUS-33
Moraceae	*Ficus sycomorus *L.	Shafo (KA) Wupur (KW)	Tree	Fruit (whole)	Raw	DUS-10
Moringaceae	*Moringa stenopetala *(Bak. f.) Cuf.	Haleko (KA) Kalenko (KW)	Tree	Leaf	Vegetable, boiled	DUS-09
Nymphaeaceae	*Nymphaea lotus *L.	Kutako	Herb	Root	Boiled (famine food)	LEB-29
	*Nymphaea nouchawi *L.	Bodo	Herb	Root (peeled)	Boiled (famine food)	LEB-30
	.	Bodo	Herb	Seed (flower)	Flour to make bread	LEB-30
Olacaceae	*Ximenia americana *L.	Mekela (KA) Niraw (KW)	Shrub	Fruit (pulp)	Ripe, succulent	MUR-54
Salvadoraceae	*Dobera glabra *(Forssk.) Poir.	Shala (KA) Shelada (KW)	Tree	Fruit (whole)	Ripe, boiled (famine food)	DUS-64
	*Salvadora persica *L.	Mero	Shrub	Fruit (whole)	Raw juice (famine food)	MUR-46
Sapindaceae	*Lecaniodiscus fraxinifolius *Bak.	Choro (KA) Eyoli (KW)	Tree	Fruit (whole)	Boiled (porridge), sweetening	MUR-50
	*Allophylus macrobotrys *Gilg.	Belcho (KW)	Shrub	Fruit (whole)	Ripe and raw (famine food)	MUG-16
Solanaceae	*Solanum nigrum *L.	Tsepo	Shrub	Leaf	Vegetable, boiled, famine vegetable	LEB-2
	*Lycium shawii *Roem. & Schult.	Doreda	Tree	Leaf	Vegetable, boiled famine vegetable	MUR-59
Tiliaceae	*Corchorus olitorius *L.	Sisilko (KW)	Herb	Leaf	Vegetable, boiled	LEB-63
	*Grewia bicolor *Juss.	Bereza	Tree	Fruit (whole)	Raw	MUR-31
	*Grewia kakothamnos *K. Schum.	Demak	Shrub	Fruit (whole)	Raw, juice, fried, famine food	MUR-29
	*Grewia villosa *Willd.	Rug Rug (KA) Gergicha (KW)	Shrub	Fruit (whole)	Raw, juice or sweetening	DUS-28
Ulmaceae	*Celtis africana *Burm. f.	Danga (KA) Hay/Dana (KW)	Tree	Fruit (pulp)	Raw, succulent part	LEB-10
	*Celtis toka *(Forssk.) Hepper & Wood.	Zuguay (KA) Lompo (KW)	Tree	Fruit (whole)	Raw	LEB-15
Vitaceae	*Cyphostemma adenocaule *(A. Rich.) Wild & Drummond	Okoto (KA) Dumpesha (KW)	Herb	Root (peeled)	Boiled	MUG-9

**Table 2:** Univariate analysis of species richness of wild edible plants reported by Kara and Kwego people

Source	Type III Sum of Squares	df	Mean Square	F	**Sig**.
Corrected Model	4.408	45	0.098	1.069	0.431
Intercept	48.840	1	48.840	532.801	0.000
Locality	0.046	1	0.046	0.501	0.484
Family	2.283	22	0.104	1.132	0.370
Locality * Family	2.072	22	0.094	1.028	0.465
Error	2.750	30	0.092		
Total	68	76			
Corrected Total	7.158	75			

**Figure 2:**
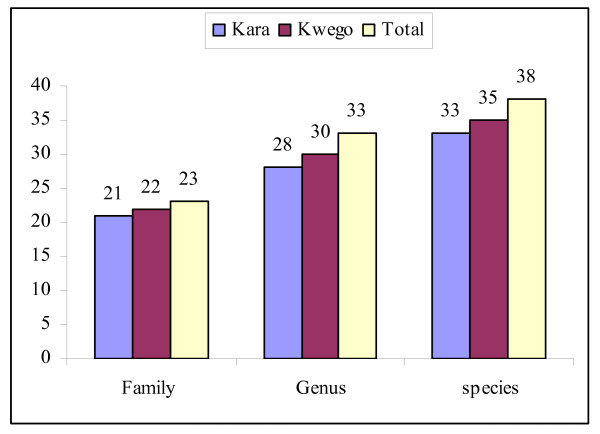
**Taxonomic diversity of wild edible plants reported by Kara and Kwego people**.

The wild edible plants species were distributed among 23 families and 33 genera (Fig. 2). The families Capparidaceae and Tiliaceae had four species each; Boraginaceae, Convolvulaceae, Cucurbitaceae, Fabaceae, Salvadoraceae, Sapindaceae, Solanaceae and Ulmaceae had two each. The remaining families had one species each. One plant was only identified to a genus level. The number of plants reported in the study area is comparable with those reported elsewhere [1,9,10,12].

### Growth forms, edible plant parts and time of gathering

**Figure 3:**
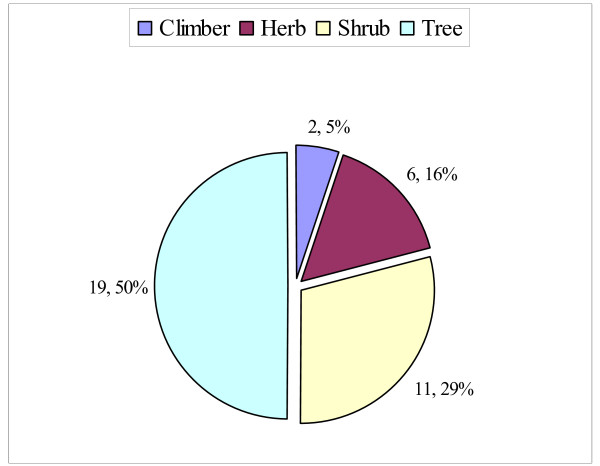
**Growth forms of wild edible plants reported in the study area, Kara and Kwego**.

**Figure 4:**
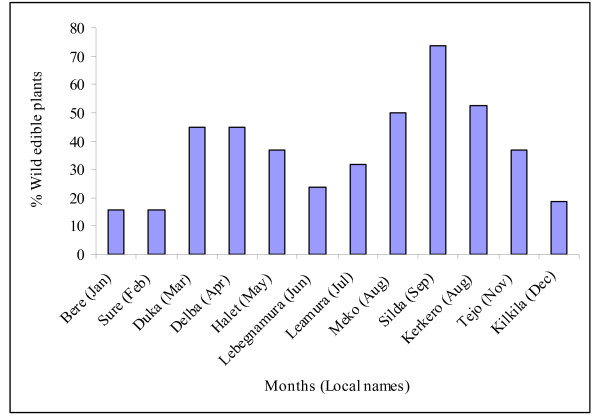
**Percent of wild edible plants consumed and time of gathering within a year, Kara and Kwego**.

Half of the reported plants, 19 (50%), were trees and two (5.26%) were climbers (Fig. 3) and there was no difference between Kara and Kwego in growth forms of the reported wild edible plants (Pearson's Chi-square test _(d.f. = 3) _= 0.872). Forty plant parts were indicated as food sources: 23 (58.97%) fruits, 13 (33.33%) leaves, 3 (7.69%) roots and one (2.56%) seed and there was no difference between Kara and Kwego people in number of wild edible plant parts used (Pearson's Chi-square test _(d.f. = 3) _= 0.994). The edible plant parts were gathered from the wild at different time of the year (Fig. 4) and the majority were gathered and consumed from *'Duka*. (March) to *'Halet*. (May) and from *'Meko*. (August) to *'Tejo*. (November). These two durations are rainy seasons at the study areas where most of the plants flower and fruit. The main rainy season is between March and May and a smaller one is between September and November [2,10,19].

Over 70% of the wild edible plants were consumed during times of food scarcity and starvation from *'Leamura' *(July) to *'Kilikila' *(December) where the stored cultivated food crops are dwindling progressively. These plants are used as substitutes and fill the gap of food deficiency that happens between harvesting seasons [3,5,8,25]. *Diospyros mespiliformis *Hochst.ex A. DC., *Grewia villosa *Willd., *Maerua subcordata *(Gilg.) De Wolf, *Maytenus senegalensis *(Lam.) Exell, and *Fabaceae sp*. were usually gathered and consumed from February to May where the cultivated food crops are in plenty.

### Edible plants used as vegetables

Sixteen (41%) wild edible plants were used as vegetables by both groups. Of these, thirteen were harvested for their leaves or young twigs or upper parts (leaf and stem) and three were harvested for their fruits (Table 1). All plant parts used as vegetables were gathered from the surrounding forest, and they were chopped and boiled to *'Belesha' *(sauce) or used as relishes to 'Adano' (porridge). The leaves of *Leptadenia hastala *(Pers.) Decne., *Cadaba forinosa *Forssk., *Ipomoea plebeia *R. Br., and *Moringa stenopetala *(Bak. f.) were used throughout the year as a regular vegetable though the leaves of the last two plants are repeatedly boiled to be edible. *Moringa stenopetala *(Bak. f.) Cuf is used as leaf vegetable in southwest Ethiopia and in the territory bordering Kenya [8,10,26]. The remaining nine leaf vegetables were consumed as substitutes of cultivated vegetables: 'Wuhawaka' (*Vigna unguiculata *(L.), Ketele (*Vigna vexillata *(L.) A. Rich) and 'Botolo' (*Cucurbita pepo *L.) during the dry season and during times of scarcity or famine. They provide the major vitamins and minerals for much of the year [10,27,28]. The ripen fruits of *Coccinia grandis *(L.) Voigt. *Diospyros mespiliformis *Hochst.ex A. DC. and *Flueggea virosa *(Willd.) Voigt. were used as vegetables. The fruits are boiled to make sauces or are cooked together with the powder of other cultivated crops to make porridge [10].

The vegetables were collected and prepared by women, but consumed by both genders and all age groups. In other parts of Ethiopia, *Corchorus olitorius *L. and *Cleome gynandra *L. are used as supplementary foods, and *Solanum nigrum *L. is used as a substitute for cultivated vegetables at time of scarcity [8-10,12,19,22].

### Wild edible fruit plants

Nineteen (50%) were wild edible fruits gathered when ripened at different times of the year and consumed raw or boiled. Four of the edible fruits were used to make juices mixed with water or boiled to make sauces as a relish to porridge. *Lecaniodiscus fraxinifolius *Bak., *Saba comorensis *(Boj.) Pichon, *Salvadora persica *L., *Balanites rotundifolia *(van Tieghem) Blatter, *Tamarindus indica *L., *Dobera glabra *(Forssk.) Poir., *Uvaria leptocladon *Oliv., and *Celtis toka *(Forssk.) Hepper & Wood. were consumed from *'Leamura' *(July) to *'Kilikila' *(December) when there is a severe food shortage. Mostly children and herdsmen gather and consume fruits at times of the year when food is plentiful, but both genders and all age groups gather and consume these during food shortages and times of starvation as in other parts of Ethiopia and elsewhere [8-10,19,20].

Some of the wild edible plants reported in this study are also consumed in other parts of Ethiopia in times of plenty and food scarcity: *Balanites rotundifolia *(van Tieghem) Blatter, *Borassus aethiopum *Mart, *Cadaba farinosa *Forrsk., *Cleome gynandra *L., *Coccinia grandis *(L.) Voigt., *Corchorus olitorius *L., *Cordia sinensis *Lam., *Diospyros mespiliformis *Hochst.ex A. DC., *Dobera glabra *(Forssk.) Poir., *Ficus sycomorus *L., *Flueggea virosa *(Willd.) Voigt., *Grewia bicolor *Juss., *Grewia villosa *Willd, *Kedrostis foetidissima *(Jacq.) Cogn., *Leptadenia hastala *(Pers.) Decne., *Maerua subcordata *(Gilg) De Wolf, *Moringa stenopetala *(Bak. f.) Cuf., *Saba comorensis *(Boj.)Richen, *Salvadora persica *L., *Solanum nigrum *L., *Tamarindus indica *L., *Uvaria leptocladon *Oliv., and *Ximenia americana *L. [8-10,19,20] .

### Edible root and seed plants

The edible roots of *Nymphaea lotus *L., *Nymphaea nouchawi *L. and *Cyphostemma adenocaule *(A. Rich.) Wild & Drummond were gathered and consumed during times of food shortage and famine (Table 1). The roots, excluding the peel, were boiled and consumed as a whole meal by both gender and all age groups. The seeds of *Nymphaea nouchawi *L. were collected from dried flowers of the plants, and the flour was used to make bread. Besides the underground parts, the leaves of *Cyphostemma adenocaule *(A. Rich.) Wild & Drummond are also used as vegetable in other parts of Ethiopia [8,10].

### Medicinal and other uses of wild edible plants

Thirty-seven percent of the reported wild edible plants were also used as medicine (Table 3). Some of the plant parts used as a food source were also ingested as a remedy: *Saba comorensis *(Boj.) Pichon, *Moringa stenopetala *(Bak. f.) Cuf., *Ximenia americana *L. and *Grewia bicolor *Juss. Most of the medicinal plants were trees and shrubs, and roots were predominantly used as a remedy The use of root as medicine may have a serious consequence from both an ecological point of view and from the survival of the wild edible plants since there are no home gardens that could play a role in easing harvest from the forest and conservation of medicinal plants [29,30].

**Table 3:** List of wild edible plants used as remedy, Kara and Kwego people

Family	Species	Plant part used	Medicinal Uses	Preparation
Anonaceae	*Uvaria leptocladon *Oliv.	Root	Respiratory infection and tuberculosis	Crushed/decoction
Apocynaceae	*Saba comorensis *(Boj.) Pichon	Fruit	Venereal disease/syphilis	Powder with water
Balanitaceae	*Balanites rotundifolia *(Van Tiegh.) Blatter	Root	Gastro intestinal illness and intestinal parasites	Crushed boiled with goat meat
Boraginaceae	*Cordia sinensis *Lam.	Root	Respiratory infection and tuberculosis	Crushed/infusion
Capparidaceae	*Cadaba forinosa *Forssk.	Root	Gastro intestinal illness and intestinal parasites	Chewing
Euphorbiaceae	*Flueggea virosa *(Willd.) Voigt.	Root	Boils, abscess and swelling	Crushed/decoction
Moringaceae	*Moringa stenopetala *(Bak. f.) Cuf.	Leaf	Headache and flu	Boiled soup
Olacaceae	*Ximenia americana *L.	Fruit (young)	External injury and wounds	Squeezed on the site
Salvadoraceae	*Dobera glabra *(Forssk.) Poir	Root	Respiratory infection and tuberculosis	Crushed/infusion
	*Salvadora persica *L.	Root	Respiratory infection and tuberculosis	Crushed/decoction or boiled with goat meat
Solanaceae	*Lycium shawii *Roem. & Schult.	Root	Boils, abscess and swelling	Crushed/decoction/boiled with goat meat
Tiliaceae	*Grewia bicolor *Juss.	Fruit	Venereal disease/syphilis	Soaked in water
	*Grewia kakothamnos *K. Schum.	Root	Respiratory infection and tuberculosis	Crushed/decoction
	*Grewia villosa *Willd.	Bark	Boils, abscess and swelling	Crushed/infusion

Ten (23.6%) of the reported wild edible plans were used for other functions: *Grewia bicolor *Juss. was used for making *Napala*, a prestigious decorated stick used by respected elders; *Celtis africana *Burm. f. for making *haypeti*, a stick used for digging to plant seeds and for fishing; *Grewia kakothamnos *K. Schum. for making *omo*, a bow for arrow; *Diospyros mespiliformis *Hochst.ex A. DC. for making *burkuta*, traditional small three legged seat that also serves as a pillow, which is used only by elderly men and women are totally forbidden; *Maerua subcordata *(Gilg.) De Wilf for water purification; *Maerua oblongifolia *(Forssk.) A. Rich. for making *shorka*, spoon and also used as teeth brush; *Ximenia americana *L., for processing goat's skin to make traditional clothing: *tudaye*, *abiray *and *kechdiay *that are worn by females; *Cordia sinensis *Lam. for making *yibet*, oar and bee hive; *Ficus sycomorus *L. for making bee hive and *lomojo*, canoe, and *Celtis toka *(Forssk.) Hepper & Wood. for making canoes. The practice of using plants with big trunks to make bee hives and canoes was observed as being destructive and a threat to wild edible plants since there are not any conservation practices employed in the localities. Bark is used to make the bee hive and the trunk is hewed to make a canoe.

## Conclusions

Over seventy percent of the wild edible plants are consumed when food scarcity is high and at times of starvation; consumption of these plants increases as the stock of cultivated crops dwindles progressively. These plants are used as substitutes and fill the gap of food deficiency. But information on the nutritional values and possible toxic effects of most of the wild edible plants reported by Kara and Kwego, and others in Ethiopia is not available. Therefore, the information documented on the wild edible plants of Kara and Kwego may serve as baseline data for future studies on nutritional values and possible side effect, and to identify plants that can improve nutrition and increase dietary diversity in the study areas and elsewhere. All plant parts used as vegetable in Kara and Kwego are gathered from the wild whilst some are grown in home gardens in other parts of Ethiopia. Hence, the data compiled in this study can assist in selection and domestication of wild vegetable plants, which are available throughout the year, to be grown in home gardens as alternative vegetable sources. Some of these wild edible plants may have the potential to be a valuable food source (if cultivated) and could be part of a strategy in tackling food insecurity.

## Competing interests

The authors declare that they have no competing interests.

## Authors' contributions

The authors have made substantive intellectual contributions to this study in data collection, identification of plants, preparation of the manuscript and proof reading. All authors have read and approved the final manuscript.
